# Comparative Evaluation of Existing and Rationally Designed Novel Antimicrobial Peptides for Treatment of Skin and Soft Tissue Infections

**DOI:** 10.3390/antibiotics12030551

**Published:** 2023-03-10

**Authors:** Anna Ramata-Stunda, Martins Boroduskis, Elza Kaktina, Liene Patetko, Uldis Kalnenieks, Zane Lasa, Marta Rubina, Inese Strazdina, Gints Kalnins, Reinis Rutkis

**Affiliations:** 1 Alternative Plants Ltd., 2 Podraga Str., LV-1007 Riga, Latvia; 2 Laboratory of Bioanalytical and Biodosimetry Methods, Faculty of Biology, University of Latvia, 3 Jelgavas Str., LV-1004 Riga, Latvia; 3 Institute of Microbiology and Biotechnology, University of Latvia, 1 Jelgavas Str., LV-1004 Riga, Latvia; 4 Latvian Biomedical Research and Study Centre, 1 Ratsupites Str., LV-1067 Riga, Latvia

**Keywords:** antimicrobial peptides, cytotoxicity, skin pathogens, acne

## Abstract

Skin and soft tissue infections (SSTIs) and acne are among the most common skin conditions in primary care. SSTIs caused by ESKAPE pathogens (*Enterococcus faecium*, *Staphylococcus aureus*, *Klebsiella pneumoniae*, *Acinetobacter baumannii*, *Pseudomonas aeruginosa*, and *Enterobacter* sp.) can range in severity, and treating them is becoming increasingly challenging due to the growing number of antibiotic-resistant pathogens. There is also a rise in antibiotic-resistant strains of *Cutibacterium acne*, which plays a role in the development of acne. Antimicrobial peptides (AMPs) are considered to be a promising solution to the challenges posed by antibiotic resistance. In this study, six new AMPs were rationally designed and compared to five existing peptides. The MIC values against *E. coli*, *P. aeruginosa*, *K. pneumoniae*, *E. faecium*, *S. aureus*, and *C. acnes* were determined, and the peptides were evaluated for cytotoxicity using Balb/c 3T3 cells and dermal fibroblasts, as well as for hemolytic activity. The interaction with bacterial membranes and the effect on TNF-α and IL-10 secretion were also evaluated for selected peptides. Of the tested peptides, RP556 showed high broad-spectrum antibacterial activity without inducing cytotoxicity or hemolysis, and it stimulated the production of IL-10 in LPS-stimulated peripheral blood mononuclear cells. Four of the novel AMPs showed pronounced specificity against *C. acnes*, with MIC values (0.3–0.5 μg/mL) below the concentrations that were cytotoxic or hemolytic.

## 1. Introduction

There are numerous species of bacteria colonizing the skin and forming normal microflora. When skin barrier is broken, these, including opportunistic pathogens, can invade into the tissue, moreover compromised skin barrier poses a high risk for tissues to get infected with non-opportunistic pathogens [1,2]. Skin and soft tissue infections (SSTI) are among the most common bacterial infections in primary care and can often lead to hospital admissions. SSTIs vary in severity, invasiveness, and causative agents. They range from mild infections to deep abscesses and necrotizing fasciitis [3]. Infections can be monomicrobial and polymicrobial. In the majority of cases, Gram-positive bacteria dominate in SSTIs, with *Staphylococcus aureus* and *Streptococci* being the most common causative agents [4]. When more than one species of bacteria is involved in SSTIs there can be a synergistic effect, that increases the pathogenicity and present further challenges for infection management. The class of microorganisms known as ESKAPE (*Enterococcus faecium*, *Staphylococcus aureus*, *Klebsiella pneumoniae*, *Acinetobacter baumannii*, *Pseudomonas aeruginosa*, and *Enterobacter* sp.) are among the most prevalent bacteria in cutaneous polymicrobial infections [5,6].

Antibiotics are widely used to treat SSTIs. For antibiotic therapy to be effective it should not only have bactericidal effect, but also have the ability to penetrate the skin tissue. Majority of the treatments for SSTI infections are topical, with systemic antibiotics being used only in severe cases [5,6]. The choice of antibiotic therapy might be challenging and is highly dependent on the nature of the infection and the bacteria present. For example, abscesses caused by ESKAPE pathogens are difficult to treat and are often highly resistant to antibiotics. Frequent high doses of broad spectrum antibiotics for prolonged periods are often prescribed for such cases [7]. The number of antibiotics effective against ESKAPE pathogens is declining, and there is lack of new effective therapeutics [8,9].

Antibiotic resistance is named by World Health Organization (WHO) as one of the top 10 global health threats and it is already listed as one of the leading causes of death worldwide [10]. In 2019, it was already estimated that 4.95 million deaths were associated with antibiotic resistance [11]. Antibiotic resistance, including multidrug resistance, is widespread in *Escherichia coli* [12,13], *Staphylococcus aureus* [14], *Klebsiella pneumoniae* [15], *Streptococcus* spp. [16], *Acitenobacter baumanii* [17], and *Pseudomonas aeruginosa* [18,19,20].

Another skin disease, the pathogenesis of which is associated with proliferation and invasion of bacteria, is acne. Acne is not considered a typical infectious disease, however bacteria play an important role in its development. Acne is the most common skin disease worldwide frequently affecting adolescents but also increasing in incidence among adults [21,22,23]. *Cutibacterium acnes* (previously *Propionibacterium acnes*) is a human skin commensal and opportunistic pathogen that causes acne. Different phylotypes of *C. acnes* are found in healthy and acne prone skin. *C. acnes* affects keratinocyte and fibroblast differentiation, promotes inflammatory processes leading to acne lesions, and promotes sebum production and the development of comedones. Topical antibiotics are widely used for treatment of acne, in severe cases systemic antibiotics are prescribed. Due to the wide use of antibiotics and long treatment courses used for acne, resistance of *C. acnes* is a growing problem with numbers of multiresistant strains raising. Limited number of non-antibiotic treatments are available for acne. Benzoyl peroxide, azelaic acid, salicylic acid, or topical retinoids are often used alone or in combination with antibiotics with varying success [24,25,26,27,28].

Skin infections caused by antibiotic-resistant bacteria are of a higher risk of invasive disease and failure of the treatment. To overcome challenges associated with the treatment of skin infections caused by resistant bacteria, new antimicrobial therapeutics are searched for. Currently, in early stage clinical studies, there are various host defense proteins, synthetic and semisynthetic antibiotics, lysins, monoclonal antibodies, antibody-antibiotic conjugates, and non-peptide chemicals mimicking antimicrobial peptides [29,30].

Various antimicrobial peptides (AMPs) have been investigated for their potential applications in SSTI and acne therapies. AMPs are short host defense oligopeptides produced by all kingdoms of living organisms. In nature, they are part of the innate immune system. AMPs can be synthesized, produced biotechnologically, or isolated from natural sources. AMPs are classified according to their secondary structure with β-sheet and α-helix structures being the most common. AMPs’ mechanisms of action are considered to be non-specific which broadens their spectrum of activity and allows to target antibiotic resistant strains [31]. The antibacterial activity of AMPs mainly depends on their ability to interact with the pathogen’s cytoplasmatic membrane, cell wall, or intracellular targets (e.g., nucleic acid or protein synthesis). Targeting membranes allows AMPs to kill metabolically active as well as slowly growing and dormant cells. It is considered that due to AMPs’ rapid action and non-specific targets, microorganisms are unable to develop resistance [32,33,34]. However, in isolated cases, resistance to naturally occurring peptides has been observed. Mechanisms behind bacterial resistance to AMPs are remodeling bacterial surfaces, proteolytic degradation of AMPs by bacterial enzymes, and efflux pumps capable of expelling AMPs [35]. To overcome risk of resistance derivatives of natural AMPs and synthetic AMPs are developed. Antimicrobial activity depends on the amino acid composition and structure of AMPs. Even minor changes can result in considerable differences in the activity of similar peptides [36]. AMPs have high affinity for negatively charged bacterial membranes, yet they often exhibit undesired effects as cytotoxicity and hemolysis in mammalian systems [37]. Overcoming toxicity while maintaining high antibacterial activity is one of the challenges in development of novel AMPs.

Until today, several AMPs have been shown to have promising activity and safety profile in both in vitro and in vivo studies. At the same time, the number of clinically approved AMPs remains low [9]. Several AMPs have been tested specifically for their efficacy against ESKAPE pathogens. Among them is histatin 5, a natural human salivary peptide, petide LL-37 naturally produced by epithelial and immune cells, a synthetic cationic peptide, and human ApoB derived peptides. On top of being bactericidal, these peptides also possess antibiofilm activity [9,38,39]. Peptide LL-37 and its derivatives have been studied for topical applications to treat wounds infected with *P. aeruginosa* and *S. aureus*. In wounds, these bacteria produce biofilms that are less susceptible to antibiotic therapy. Apart from having bactericidal and antibiofilm activities, AMPs can be chemotactic for immune cells, modulate the expression of pro and anti-inflammatory cytokines, regulate cell proliferation and differentiation, stimulate angiogenesis, and interact with lipopolysaccharide [40,41,42,43,44].

In an in vitro study, granulysin-based peptides were effective in killing *C. acnes* and also reduced the release of pro-inflammatory cytokines [32,45]. Anti-acne and immunomodulating activities have been reported for synthetic AMPs based on natural AMPs from bacteria, frogs, and cows [46,47]. LZ1 peptide developed through fusion of known families of antimicrobial peptides is effective against *C. acne*, *S. epidermidis*, and has anti-inflammatory activity [48]. Another example is a bromine-containing peptide derived from sea urchins with activity against *C. acnes* exceeding that of antibiotics clindamycin and erythromycin [49]. Several other peptides naturally produced by human keratinocytes and immune cells have been investigated for their potential to inhibit *C. acnes* [46,47]. 

AMPs could be used in monotherapy or in combination with antibiotics. In combination, action of AMPs could increase the penetration of antibiotics that have intracellular targets. Such approach had been demonstrated in murine in vivo model of infection with ESKAPE pathogens [7]. Synergistic activity might also allow the use pf lower AMP concentrations and escape AMP cytotoxicity. 

Current data on bactericidal activity, along with limited ability of bacteria to develop resistance to AMPs clearly points to the potential of AMPs to be developed for various therapeutic applications. By combining almost unlimited sequence options with accumulated data on the structural and functional properties of the existing AMPs, AMPs can be rationally designed to meet desired biological parameters and thus turned into novel therapeutics. In this study, novel AMPs were rationally designed and tested for their activity against selected bacteria involved in pathogenesis of skin infections, and their potential applicability evaluated based on cytotoxicity and hemolytic activity. 

## 2. Results

### 2.1. Antimicrobial Peptides

The sequences of all peptides used in this study are presented in Table 1. Five AMPs were examined earlier for treatment of individual bacteria related to skin and soft tissue infections, while none of them were tested against all ESCAPE pathogens simultaneously. Peptide R1 is a direct derivate of earlier published RP551 [47], with the replacement of non-proteinogenic amino acid, ornithine (O) by lysine (K) at the positions 3,8,11, and 14. According to Woodburn et al., 2020 [47] and Zhang et al., 2013 [48], RP551, RP556 and LZ1 peptides possesses antimicrobial activity against *C. acnes* at 2–4 μg/mL and <1 μg/mL scale, respectively. AA139 was selected as it possesses activity against multidrug-resistant Gram-negative bacteria [50], and PA13 was selected because it is a rationally designed, hybrid AMP, inspired by cathelicidin and aurein with reported activity (MIC 4–16 μg/mL) against *P. aerugiosa* [51]. Oligopeptide 10 (Oligo10) was selected for reference as it is one of few commercially available AMPs widely used as an active ingredient in skincare products to cope with various skin and soft tissue conditions. Remaining 6 AMPs (R1, R10-R14) are novel peptides rationally designed within this study. The design of the novel peptides was inspired by known natural and synthetic AMPs, adhering to a general principle of being amphipathic and positively charged. Hydrophobic amino acids such as phenylalanine and tryptophan were directed towards one side, while positively charged lysines were directed towards the other side of the AMPs in order to achieve the desired structure and sequence.

### 2.2. Antibacterial Activity

Comparative evaluation of the antimicrobial activity of all AMPs against library of pathogens associated with skin infections revealed that PR556 and R10 had the highest overall antibacterial activity (Table 2). RP556 was the most active against Gram-positive *S. aureus* and *E. faecium*. Low concentrations (MIC 2–4 μg/mL) of RP556, R10, and R13 inhibited *E. coli*. RP556 and R10 had similar activity against *P. aeruginosa* (MIC 2 μg/mL). R13, R13, R14 inhibited *P. aeruginosa* at concentration 4 μg/mL. *K. pneumoniae* was most susceptible to peptides R1, RP556 and R14. Most peptides, except AA139, LZ1, and PA13 inhibited *C. acnes* at concentration 2 μg/mL and below. R11 and R13 had the lowest MIC values against *C. acnes*. R11 might be hypothesized to be highly specific for *C. acnes*, as its MIC values against all other bacteria were above 16 μg/mL. AA139 was the least effective against *C. acnes* with MIC value of 63 μg/mL. Oligo10 and R11 were the least effective against Gram-negative bacteria. Data on sensitivity of bacteria to positive controls (antibiotics and non-antibiotic antimicrobial agents) are provided in Supplementary material (Table S1).

No clear correlations between antibacterial activity and secondary structure or peptide size were observed.

### 2.3. Cytotoxicity

**Figure 1:**
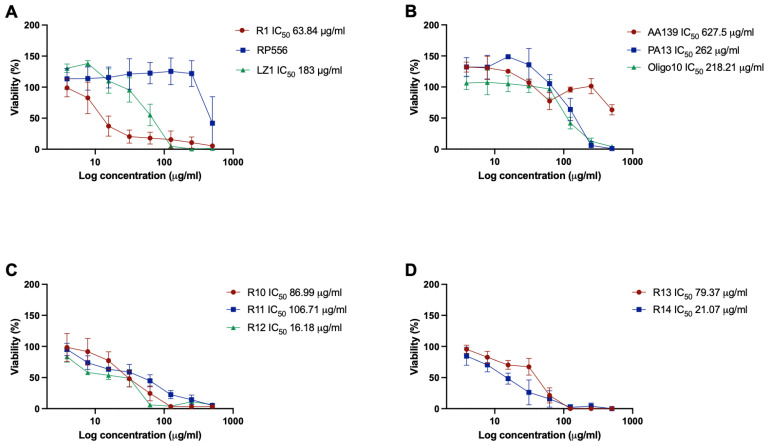
Viability of Balb/c 3T3 cells after incubation with antimicrobial peptides. (**A**) peptides R1, RP556, LZ1, (**B**) peptides AA139, PA13, Oligo10, (**C**) peptides R10, R11, R12, (**D**) peptides R13, R14. Viability expressed as %, compared to control, where applicable IC_50_ values are shown, *n* = 5.

Cytotoxicity screening in the Balb/c 3T3 cell line showed that peptides vary considerably in their effect on cell viability. There was no correlation between cytotoxicity and secondary structure of peptides. Peptide RP556 (Figure 1A) was the least cytotoxic—negative effect on cell viability was observed only at the highest concentration. Moreover, in the presence of RP556 at concentrations 3.9–250 μg/mL, a slight increase in cell proliferation was observed as viable cell count raised on average by 19.15 ± 4.76%. Peptide R1 was not cytotoxic at 3.9 and 7.8 μg/mL level (changes in cell viability did not exceed 20%). These are concentrations that are close to or above MIC values against most of the selected microorganisms, except *E. faecium*. At concentration 15.63 μg/mL and above R1 was cytotoxic and decreased cell viability by more than 62%. Reduced cell viability was observed in presence of LZ1 at concentrations above 62.5 μg/mL, clearly indicating that MIC values are well below cytotoxic levels. AA139 was not cytotoxic at concentrations up to 250 μg/mL, the highest concentration (500 μg/mL) reduced cell viability by 36% (Figure 1B). PA13 was cytotoxic at concentrations above 125 μg/mL. Similar to RP556 peptides, AA139 and PA13 at specific concentration ranges had positive effects on cell proliferation. AA139 increased viable cell counts by 29.46 ± 3.57% at concentration level 3.9–15.63 μg/mL, and PA13 by 37.15 ± 7.89% at concentration range 3.9–31.25 μg/mL. Oligo10 was cytotoxic at concentrations starting from 125 μg/mL. Low concentrations (3.9–7.8 μg/mL) of R10 and R13 (Figure 1C,D) were nontoxic for cells, while higher concentrations drastically reduced cell viability. For R11, R12, and R14, only the lowest tested concentration (3.9 μg/mL) did not have a negative effect on cell viability.

Using data acquired in antimicrobial activity and cytotoxicity assays, therapeutic indexes (TI) were calculated for preliminary assessment of clinical applicability of AMPs. In general, the higher the TI, the greater the specificity of AMPs to inhibit bacterial pathogens without negative effect on mammalian cells. RP556 had the TIs against Gram-positive *E. faecium* and *S. aureus*, and Gram-negative *P. aeruginosa* and *K. pneumoniae (*Table 3). Peptides RP556, R10, R11, and R13 had high indexes against *C. acnes*. At the same time, these peptides, except for RP556, had low TIs against all other bacteria included in the study. This result allows us to speculate that peptides R10, R11, and R13 could be used at low concentrations to specifically target *C. acnes* without having effect on other commensal microorganisms of skin.

**Figure 2:**
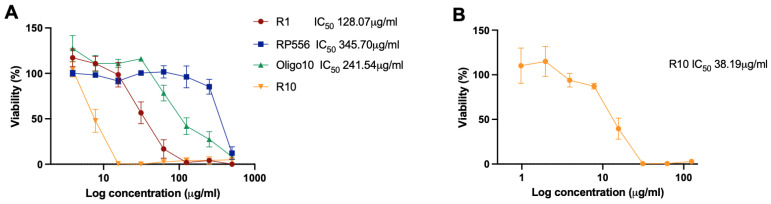
Viability of human primary dermal fibroblasts after incubation with antimicrobial peptides. (**A**)—peptides R1, RP556, Oligo10, and R10 at concentration range 3.9–500 μg/mL), (**B**)—peptide R10 at concentration range 0.98–125 μg/mL). Viability expressed as %, compared to control, *n* = 3.

Selected AMPs were tested in human primary dermal fibrobast (DF) cell cultures (Figure 2). It was found that R10 was more cytotoxic in DF than Balb/c 3T3 cell cultures, while RP556 and Oligo10 had similar cytotoxicity profiles in both cell lines, and R1 was less cytotoxic in DF. In DF cell culture, RP556 did not show stimulating activity as in Balb/c 3T3 cells, but Oligo10 at the lowest concentration (3.9 μg/mL) slightly increased viable cell counts by 27.34 ± 14.22%.

TIs for the selected AMPs were calculated using the cytotoxicity assessment in DF (Table 4). Similarly, as in the case of Balb/c 3T3, in DF cultures the highest overall TIs were obtained for RP556. R1, Oligo10, and R10 had comparably low TIs, except against *C. acnes.* This again allows for speculation that low concentrations of selected AMPs might be specific for *C. acne* but not for other commensal or pathogenic bacteria.

### 2.4. Hemolysis

**Figure 3:**
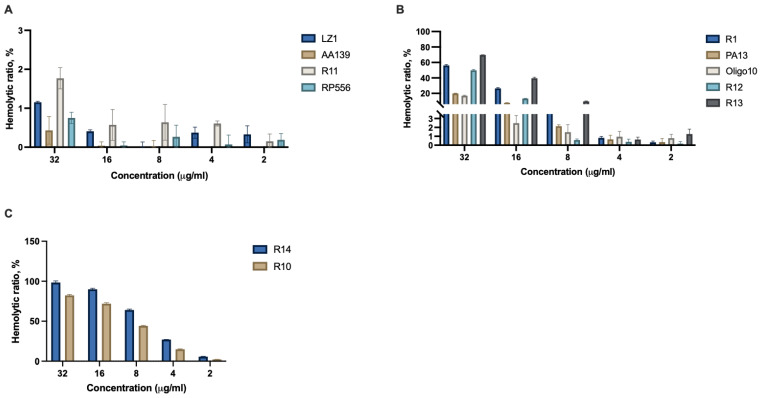
Hemolysis of fresh human blood by antimicrobial peptides AA139, LZ1, R11, R2 (**A**), R1, PA13, Oligo10, R12, R13 (**B**) and R14m R10 (**C**). *n* = 3.

Hemolysis assay was performed using fresh human blood to evaluate the potential of antimicrobial peptides to induce red blood cell lysis. Peptides LZ1, AA139, R11, and RP556 did not induce hemolysis (Figure 3A); within the concentration range, the 2–32 μg/mL hemolysis ratio (HR) was below 2%, which is considered to be an acceptable level for clinical applications [53]. For R11, additionally, 128 and 64 μg/mL were tested, and only mild hemolytic activity was detected; HRs were in the range of 2.85–7.03%. Peptides R1 and R13 exhibited strong hemolytic activity at concentration range 8–32 μg/mL, PA13 and R12 were hemolytic at 32 and 16 μg/mL, and Oligo10 induced hemolysis at 32 μg/mL and showed mild hemolytic activity at 16 μg/mL (Figure 2B). Peptides R14 and R10 demonstrated strong hemolytic activity (Figure 2C). R14 was hemolytic at all tested concentrations (HR > 5%), and R10 at 2 μg/mL had minor hemolytic activity (2.26%). Additionally, for R10, 0.5 and 1 μg/mL concentrations were tested and no hemolysis was detected (HR 0.2–0.4%).

Correlation between cytotoxicity and hemolysis was poor. Of peptides RP556, AA139, neither had pronounced cytotoxicity, nor induced hemolysis. LZ1 and R11 at higher concentrations had negative effects on Balb/c 3T3 viability, however, they did not show hemolytic activity. PA13 was cytotoxic only at high concentrations, however it induced hemolysis at a much lower concentration range. R10 and R11 had similar cytotoxicity profiles, however they differed considerably in their hemolytic activity. R12, R14, and RP551 were the most cytotoxic among all tested peptides, but their effects on red blood cells varied. R14 showed the most pronounced hemolytic activity, while RP551 did not induce hemolysis, and R12 has hemolytic only above 16 μg/mL.

### 2.5. Immunomodulatory Activity

Peptides RP556 and R10, due to their high antimicrobial activities, were chosen for evaluation in peripheral blood mononuclear cell (PBMNC) cultures. The effect on secretion of pro- and anti-inflammatory cytokines was tested, as the immunomodulatory activities of antimicrobial medicines are of particular interest in order to add complementary benefits alongside their antipathogenic activity.

**Figure 4:**
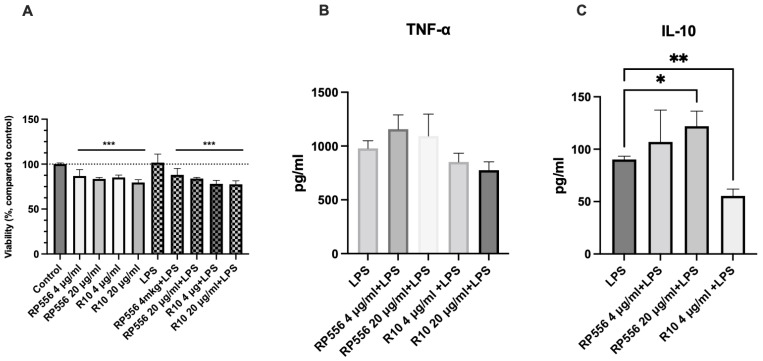
Evaluation of peptides’ effect on peripheral blood mononuclear cell (PBMNC) viability (**A**) and secretion of TNF-α (**B**) and IL-10 (**C**). LPS—lipopolysaccharide, control—cells incubated under normal conditions; *n* = 3, * *p* < 0.05, ** *p* < 0.01, *** *p* < 0.001.

Given the different levels of cytotoxicity and hemolytic activity exhibited by both peptides, viability of PBMNCs after 24h incubation was assessed (Figure 4A) prior to measurement of cytokine levels. Both peptides slightly reduced viability of cells, however changes did not exceed 17.5% for RP556 and 22.4% for R10. In the case of R10, higher concentration had a more pronounced effect and combination with LPS and further reduced viability. RP556 induced secretion of TNF-α in lipopolysaccharide (LPS) stimulated PBMNCs, but in the presence of R10, TNF-α secretion decreased (Figure 4B). RP556 statistically and significantly increased secretion of anti-inflammatory cytokine IL-10. A 35.56% increase was detected in cell cultures incubated with LPS and 20 μg/mL RP55 compared to LPS alone (Figure 4C). This exceeds the change in TNF-α secretion, where RP556 produced increase below 18.3%. When 20 μg/mL R10 in combination with LPS was used, no IL-10 secretion was detected, while 4 μg/mL R10 significantly reduced IL-10 secretion. Contrary to the RP556, the effect induced by R10 was unexpected and could be considered as undesirable.

### 2.6. Localization of AMPs in Membranes

**Figure 5:**
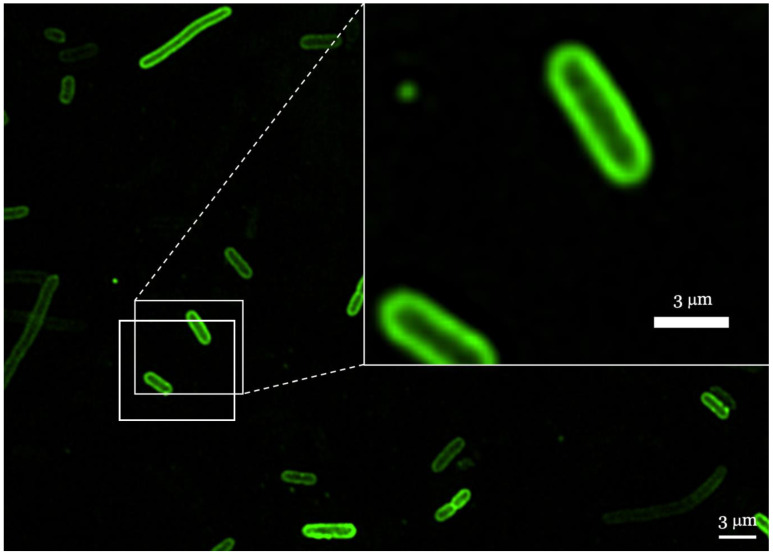
Localization of FITC-labeled RP556 peptide in *E. coli* cells imaged by confocal microscopy. The image is also representative for R1 and R10 peptides.

To investigate the localization of AMPs in membranes, peptides RP556 and R10 were labeled with Fluorescein-5-isothiocyanate (FITC, λex = 495 nm, λem = 519 nm). *E. coli* cells were incubated with FITC-labeled peptides in concentrations reaching half of their MIC values, and were observed via confocal microscopy. As shown in Figure 5, FITC-labeled RP556 peptide molecules were located at the cell membrane, indicating that this peptide can penetrate the cell membrane of *E. coli*. Likewise, R10 peptide behaved similarly, and the obtained sample images resembled the images shown in Figure 5.

**Figure 6:**
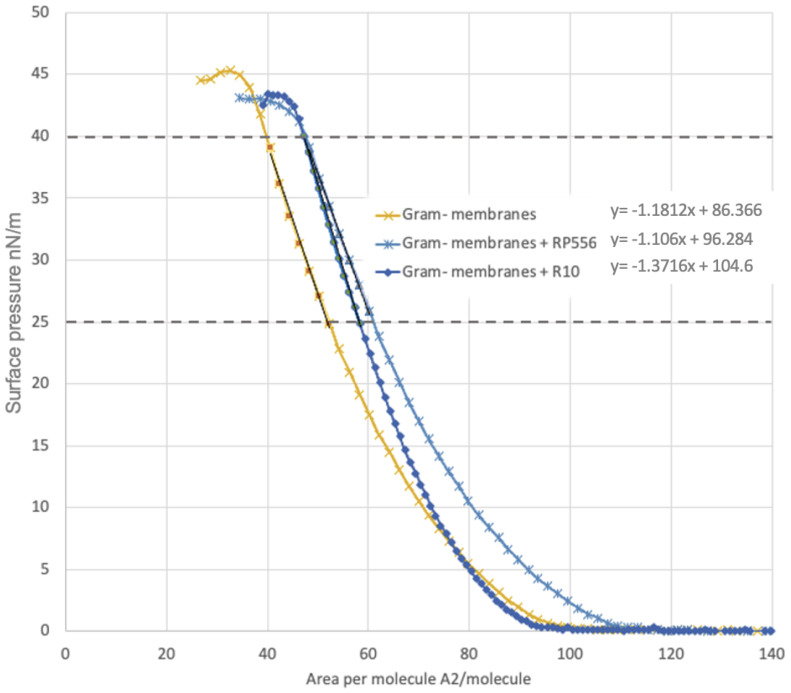
Localization of π–A isotherms for Gram-membrane model mixtures with peptides RP556 and R10.

Taken together, the interaction of FITC-labeled AMPs with *E. coli* membrane investigated by confocal microscopy revealed that both peptides can spontaneously insert into phospholipid membranes (Figure 5). However, since confocal microscope experiments involved the use of FITC fluorescent tag, we could not exclude that tagging of peptides might have somewhat altered their physiochemical properties. Therefore, membrane peptide interactions were further studied by means of the Langmuir Blodget (L-B) monolayer technique as Langmuir monolayers formed at the air/water interface. It is well established that investigations of the surface pressure via the monolayer area, or the so called π–A isotherms of such mixed lipid/antibiotics Langmuir monolayers, may provide information about the interactions between them [54]. Analysis of course of the π–A isotherm (Figure 6) confirmed that both peptides, R10 and RP556, intercalated in the Gram-negative model membrane and slightly reduced its stability manifesting as a clear decrease of the π–collapse values in both peptide samples. To quantitatively characterize membrane monolayers, we use the parameter often used in the L-B technique, limiting area per molecule (A_π→0_) determined by extrapolation of linear part of dense packed solid monolayer π–A isotherm to zero surface pressure. In our case, A_π→0_ is the limiting area per membrane-forming unit and the linear part typically corresponds to surface pressure range 25–40 mN/M. Likewise, confocal microscopy and L-B experiments confirmed the membranothropic nature of both peptides, and the results obtained revealed that R556 occupies a larger area of a membrane model than R10, as it has higher A_π→0_ (Table 5). As surface pressure (π) of 30 mN/m is generally taken to represent the lipid packing density that is equivalent to that of the outer leaflet of a cell membrane [55], the obtained model system results might be biologically relevant.

## 3. Discussion

Antibiotic resistance, driven by misuse and overuse of antibiotics, has become one of the major health threats of 21st century. Among infections that are challenging to treat due to antibiotic resistance are skin and soft tissue infections (SSTI). The high incidence of SSTI, the polymicrobial nature of these infections, the ability of skin pathogens to form biofilms, and the increasing number of multidrug-resistant bacteria prompts the search for new effective antimicrobials to use in SSTI therapy [36,56,57]. Antimicrobial peptides (AMP) are considered to be promising agents to target antibiotic resistant pathogens, as they act rapidly, are broad in spectrum and in general do not induce resistance [58]. Despite the promising nature of AMPs, only a few of them are tested in clinical trials. This is due to the local and systemic toxicity associated with many AMPs, which often outweighs their antimicrobial efficacy. Regardless of differences in cellular membranes, it is known that AMPs can be toxic to host cells. In addition, it has been observed that many AMPs have low metabolic stability and poor oral bioavailability. Because of these characteristics, it is considered that commercialization of AMPs for topical applications over systemic are more favorable. These practical considerations stimulate development of AMPs specifically for skin infections [34].

There are thousands of AMPs, both natural and synthetic discovered thus far, but still standardized approach for characterization of their safety and efficacy is lacking. Even so, little is understood about the correlation between antimicrobial activity and amino acid content and secondary structure. Although there are some data on charge and hydrophobicity requirements for AMPs to be effective against certain pathogens [59], lack of comprehensive understanding makes the design of novel AMPs challenging.

In this study, we compared antimicrobial activities and effects on mammalian cells of eleven different peptides; among them, five were previously characterized, including one commercial product, and six were rationally designed. MICs determined for all peptides varied considerably and no clear correlation with amino acid composition or structure was identified. Interestingly, peptides designed within this study all exhibited high *C. acnes* inhibitory activity, with MIC values ranging from 0.3–2 μg/mL. Our results regarding anti-acne activity of RP556 are consistent with published data. In addition, effect of RP556 on viability of Balb/c 3T3 and dermal fibroblast cells were in line with previously reported low cytotoxicity [47]. In this case, our study complements previously published characterization of RP556 peptides, proving that it has low cytotoxicity across different mammalian cell types and has strong inhibitory activity not only against *C. acnes*, but other skin pathogens as well. Despite its strong antimicrobial activity and proven interaction with bacterial membranes, we found that RP556 does not induce hemolysis. This potentially indicates high specificity of RP556 towards procaryotic membranes. Peptide LZ1 in our study showed weaker inhibitory activity against *C. acnes* compared to previously published data, while activity against *S. aureus* was identical. Differences were identified also regarding cytotoxicity—LZ1 did not have cytotoxic effect in HaCaT keratinocytes at concentrations up to 200 μg/mL., while in Balb/c 3T3 cells, a toxic effect was observed at concentration 62.5 μg/mL. At the same time, hemolytic activity was neither observed in previous studies nor in this study [48]. AA139 has been tested against various isolates of *E. coli*, *K. pneumonia*, and *P. aerugnosa*, and MIC values are slightly lower than in this study [50]. This might be explained by the different strains used in studies. Hemolytic activity, cytotoxicity, and activity against *E. coli* of peptide PA13 in our study were in line with previous characterizations [51], however susceptibility of other bacteria varied among studies. Observation that antimicrobial activity data vary between studies indicate differences in susceptibility of different strains to AMPs.

Differences in cytotoxicity and hemolytic activity among peptides were detected in this study, however, the correlation between these two indicators was poor. For example, RP556 and R10 showed the highest antimicrobial activities against selected bacteria, but their effects on red blood cells and Balb/c 3T3 and dermal fibroblast cells differed considerably. Greco et al., 2020 found human cell lines to be more resistant than erythrocytes to AMPs, and this correlates with earlier studies of AMP and hemolysis of rodent erythrocytes in which the cytotoxic effects on HaCaT keratinocytes were tested [37,60]. This emphasizes the necessity to include both cytotoxicity and hemolysis tests in screening for adequate characterization of AMPs. Usually, cell lines included in cytotoxicity assessment are either of rodent origin or cancerous cell lines (like HeLa, HepG2, HaCaT) that might have some differences from primary cells in how they respond to AMPs. For this reason, primary human dermal fibroblasts were included in this study to complement cytotoxicity data generated using Balb/c 3T3 cells. Two AMPs showed similar effects in both lines, while data of the other two differed significantly.

It has previously been reported that antimicrobial peptides might possess immunomodulatory activities [46,61]. Such bioactivity is beneficial for antimicrobial agents, as modulation of immune response during skin infections can promote healing and regeneration. This is of particular interest for treatment of acne and wound infections. In these cases, ability to modulate inflammation is crucial for successful outcome [62]. In this study, two AMPs with the highest overall antimicrobial activity, RP556 and R10, were tested for their ability to modulate secretion of proinflammatory cytokine TNF-α and anti-inflammatory cytokine IL-10 in LPS stimulated human PBMNCs. Results pointed to RP556 as potential modulator of IL-10 production. IL-10 plays an important role in wound healing as well as response to infections [63,64]. The ability of AMP to induce IL-10 production in addition to its strong antimicrobial activity would benefit the outcome of infection therapy.

Several mechanisms of action for antimicrobial peptides have been described, with interaction with bacterial membranes being the predominant mechanism. Additionally, various types of membrane interaction have been proposed [65,66]. In this study, confocal microscopy images show the localization of RP556 and R10 peptides in virtually intact bacterial membranes thus suggesting that bacterial growth is inhibited by a mechanism not directly related to compleate cell membrane disruption, but rather to pore formation via toroidal, barrel pore, or carpet mechanism.

The data generated on the effects of AMPs against different skin pathogens, along with information on cytotoxicity and hemocompatibility, is crucial for the further development of AMP-based skin infection therapies. Future steps should include testing against multidrug-resistant bacterial strains and conducting in vivo studies of infected wounds. Some of the peptides in this study showed strong antibacterial activity against *C. acnes*, but much lower activity against other bacteria. This specificity might be utilized to specifically target *C. acnes* while preserving the skin’s normal microflora, which is essential for skin homeostasis. However, questions about AMP specificity and the mechanisms behind it remain unanswered and require further study to fully understand the mode of action of these acne-specific peptides.

## 4. Materials and Methods

### 4.1. Synthesis of Antimicrobial Peptides

All AMPs, including FITC-labeled R1, RP556, and R10 peptides used in this work were synthesized in a 1mg scale by ProteoGenix, Schiltigheim, France, using solid-phase method. Peptide purity assayed by mass spectroscopy and by high performance liquid chromatography was >98%.

### 4.2. Quantification of Antibacterial Activity

Quantification of the antimicrobial resistance against various AMPs was carried out by measuring the bacterial growth in the presence of peptides at serial dilutes in 96 well plates in a microplate (96-well plate) reader Infinite^®^ M200 PRO Multimode Microplate Reader (Tecan, Maennedorf, Switzerland) according to our recent reports [67]. All bacterial strains were incubated at 32 °C for 13–18 h at 200 rpm. Optical density measurements (λ = 550 nm) were taken automatically at 10 min intervals. Antimicrobial resistance activity was quantified as the minimum inhibitory concentration (MIC)—the lowest concentration of a peptide which prevents growth of bacterial cells.

### 4.3. Confocal Microscopy

*E. coli* DH5α cells were grown to the midlogarithmic phase, harvested by centrifugation at 4000 rpm for 5 min, and washed twice with PBS buffer. *E. coli* cells (~0.3 mg/mL dry weight) were incubated with labeled AMPs according to methodology described earlier [51]. In brief, FITC-labeled R1, RP556, and R10 peptides (2 µg/mL) were incubated with cells for 30 min at 37 °C. Afterwards, the cells were washed twice with PBS to remove unbound labeled peptides. The cells were fixed with 4% paraformaldehyde for 20 min and washed twice with PBS. Localization of the peptides in the bacteria was observed under a spinning disk confocal microscope Andor BC43 (Oxford Instruments, Abingdon, UK).

### 4.4. Cell Lines and Cultivation

The BALB/c 3T3 murine fibroblast cell line was obtained from ATCC (American Type Culture Collection, Manassas, VA, USA). Primary human dermal fibroblasts were isolated by enzymatic digestion from skin biopsies in the Laboratory of Bioanalytical and Biodosimetry Methods, University of Latvia, in accordance with the approval of the Committee of Research Ethics of the Institute of Cardiology and Regenerative Medicine, University of Latvia. Cells were propagated in DMEM medium (Sigma, Irvine, UK), supplemented with 1% penicillin (100 U/mL)–streptomycin (100 μg/mL), and 10% calf serum (Sigma, St. Louis, MO, USA) for Balb/c 3T3 cell line and fetal bovine serum (Sigma, St. Louis, MO, USA) for primary dermal fibroblasts. All cultivations were performed in a humidified 5% CO_2_ atmosphere at 37 °C.

### 4.5. Cytotoxicity Assay

The cytotoxicity of the peptides was tested in the BALB/c3T3 cell line and primary dermal fibroblasts by the neutral red (NR) uptake assay. Cells were seeded in 96-well plates at a density of 5 × 10^3^ cells per well. After 24 h of incubation, peptides in a concentration range of 3.9 to 500 μg/mL were added. Dilutions were made in a cell cultivation medium. Cultivation in the presence of extracts was performed for 48 h. Afterwards, plates were washed with phosphate-buffered saline (PBS) (Sigma, Irvine, UK), and a 25 µg/mL NR solution (Sigma, Irvine, UK) diluted in 5%-fetal-calf-serum-containing-media was added. After 3 h, incubation in a humidified 5% CO_2_ atmosphere at 37 °C, the plate was washed with PBS, and the NR taken up by viable cells was extracted using desorbing fixative (50% ethanol/1% acetic acid/49% water). Absorbance at 540 nm was measured using a microplate reader Tecan Infinite^®^ 200 PRO (Tecan Group Ltd., Mannedorf, Switzerland). Cytotoxicity was expressed as a concentration-dependent reduction in the uptake of NR, compared to the untreated controls and calculated using the following equation:

$$\mathrm{Cell}\;\mathrm{viability}\;\left(\%\right)=\frac{{\mathrm{Abs}}_{540\mathrm{nm}}\left(\mathrm{treatment})-{\mathrm{Abs}}_{540\mathrm{nm}}(\mathrm{background}\right)}{{\mathrm{Abs}}_{540\mathrm{nm}}\left(\mathrm{untreated}\;\mathrm{control})-{\mathrm{Abs}}_{540\mathrm{nm}}(\mathrm{background}\right)}\times 100\%$$

### 4.6. Hemolysis Assay

A hemolysis test was performed to assess the hemocompatibility of the peptides. Blood from healthy donors was collected in Monovette vacutainers containing Ethylenediamine tetra-acetic acid (EDTA). Blood was diluted with 0.9% sodium chloride solution supplemented with 10 U/mL heparin (4:5 ratio by volume). Peptides were added to 1.5 mL vials containing fresh 0.49 mL PBS and incubated at 37 °C and 5% CO_2_ for 30 min, and 10 μL of diluted blood was added to each vial and incubated at 37 °C and 5% CO_2_ for 1 h. PBS was used as a negative control and deionized water was used as a positive control. After incubation tubes were centrifuged at 3000 rpm for 5 min, the supernatants were collected, and the absorbance was measured at a wavelength of 545 nm in a microplate reader Tecan Infinite^®^ 200 PRO (Tecan Group Ltd., Mannedorf, Switzerland).

The hemolytic ratio (HR) was calculated by the following equation:

$$\mathrm{HR}\;\left(\%\right)=\frac{\left(\mathrm{Abs}\left(\mathrm{sample}\right)-\mathrm{Abs}\left(\mathrm{negative}\;\mathrm{control}\right)\right)}{(\mathrm{Abs}\;\left(\mathrm{positive}\;\mathrm{control}-\mathrm{Abs}\left(\mathrm{negative}\;\mathrm{control}\right)\right)}\times 100\%$$

### 4.7. Immunomodulatory Activity

#### 4.7.1. Isolation of PBMNCs and Incubation with Peptides

The effect of the peptides was evaluated in human peripheral blood mononuclear cells (PBMNCs). Blood from healthy donors was collected in Monovette vacutainers containing EDTA. Blood was collected in accordance with the approval of the Committee of Research Ethics of the Institute of Cardiology and Regenerative Medicine, University of Latvia. Blood was diluted (1:2 ratio by volume) with 0.9% sodium chloride solution supplemented with 10 U/mL heparin and mononuclear cell fraction isolated by gradient centrifugation. Diluted blood samples were layered on Ficoll-Paque solution in ratio 2:1 (GE Healthcare, Chicago, IL, USA), and density gradient centrifugation was performed at 800× *g* for 20 min at room temperature in a swing-out centrifuge. Mononuclear cells containing buffy coats were aspirated and washed twice with phosphate-buffered saline and centrifuged at 600× *g* for 20 min at room temperature. The cell pellet was suspended in DMEM medium (Sigma, D6046, Irvine, UK) supplemented with 1% penicillin (100 U/mL)–streptomycin (100 μg/mL) and 10% fetal bovine serum (Sigma, St. Louis, MO, USA), and cells were seeded on 24-well plates at a density of 5 × 10^5^ cells per well and incubated at 37 °C, 5% CO_2_. Cells were allowed to adhere for 2 h prior to the addition of peptides at concentrations 4 µg/mL and 20 µg/mL, 5 µg/mL lipopolysaccharide (Sigma, St. Louis, MO, USA), or a combination of both. Cells were incubated for 24 h at 37 °C, 5% CO_2_, and incubation media were collected and stored at −80 °C for further analysis.

#### 4.7.2. Viability of PBMNCs

Viability of PBMNCs after incubation with peptides was evaluated using MTT reduction assay. Briefly, after incubation media was removed, 1 mL of 0.5 mg/mL MTT (3-[4,5-dimethylthiazol-2-yl]-2,5 diphenyl tetrazolium bromide) solution (Sigma, St.Louis, USA) in 5% serum containing cultivation media was added to each well. Plates were incubated for 2 h at 37 °C and 5% CO_2_ to allow insoluble formazan precipitates to form as a result of metabolic activity of viable cells. After incubation, media was removed and 0.5 mL DMSO added and plate incubated for 10 min at room temperature with gentle shaking to dissolve the formazan precipitate. Absorption was measured at 570 nm using Tecan Infinite^®^ 200 PRO (Tecan Group Ltd., Mannedorf, Switzerland).

Blank wells without cells were used for the background measurement. Proliferation relative to control was calculated using the equation below.

$$Proliferation\;\left(\%\right)=\frac{Abs_{570}\left(treatment)-Abs_{570}(background\right)}{Abs_{570}\left(untreated\;control)-Abs_{570}(background\right)}\times 100\%$$

#### 4.7.3. Quantification of TNF-α and IL-10

Concentrations of TNF-*α* and IL-10 secreted in cultivation media by PBMNCs were determined using enzyme-linked immunosorbent assay (ELISA). Human TNF-*α* and IL-10 DuoSet ELISA kits (RnD Systems^®^, Minneapolis, MN, USA) were used according to the manufacturer’s recommendations.

### 4.8. Langmuir−Blodgett Compression Isotherms

Langmuir−Blodgett compression isotherm measurements were carried out at 30 ± 1 °C using KSV LB through (KSV MiniMicro, Helsinki, Finlan) equipped with two mobile barriers (total area of 273 cm^2^) mounted on an antivibration table. Surface pressures were measured by platinum Wilhelmy plate with perimeter 39.24 mm according to methodology described by Hoyo et al., 2020 [68]. Surface pressure vs. area per molecule isotherms were obtained by computer controlled “step by step” method. According to this method, the membrane monolayer is compressed by a certain area step (in our case 225 mm^2^) and its relaxation has then been allowed. The end of this relaxation process was detected as the moment when surface pressure changes turned smaller than 0.02 mN/m per second, and at this moment, π was taken as an equilibrium surface pressure of the monolayer. After this, the membrane monolayer was compressed by the next step, etc., until all isotherms had been scanned and the monolayer collapse commenced.

*E. coli* membranes composed of 77% phosphatidylethanolamine (PE), ~13% phosphatidylglycerol (PG), and ~10% cardiolipin (CL) were selected as a model of Gram-negative strains to study the AMP membrane interactions. Then, 50 μL of *E. coli* model membranes (0.2 mg/mL) were carefully deposited on the deionized water subphase (27.2 MΩ·cm) surface using a Hamilton micro syringe. In experiments containing AMPs, 5 μL of peptide ethanol solution (1 mg/mL) was added to *E. coli* model membranes and carefully mixed prior to deposition on the water surface. Measurements of surface pressure (π) vs. mean molecular area (Mma) isotherms were collected 10 min later, after complete solvent evaporation. AMP membrane interactions were quantified by collapse pressure (π collapse) and condensed into a monolayer state thus limiting area per membrane and forming unit A_π→0_ respective to π = 0 mN/m. In brief, π collapse corresponds to the density of the monolayer packing above which it is not possible to increase the pressure any further. To obtain A_π→0_ values, the linear part of π–A isotherms (condensed monolayer state within the π range 25–40 mN/m) was approximately the same as linear function π = Mma* x + b where a and b were fitting constants and x isotherms coordinated, followed by calculation of limiting area value corresponding to π = 0 by equation A_π→0_ = −b/a.

### 4.9. Statistical Analysis

At least three replicates were analyzed throughout the study. Average ± standard deviation (SD) was used to express the experimental values. One-way analysis of variance (ANOVA) with Tukey’s multiple comparison test was used for statistical analysis. A *p*-value < 0.05 was considered to be statistically significant. (*—for *p* < 0.05; **—for *p* < 0.01; ***—for *p* < 0.001). Curve-fit analysis was performed to calculate IC_50_ values. GraphPad Prism 9 software was used for statistical analysis.

## 5. Conclusions

In the current study, promising biological activities were characterized for antimicrobial peptide RP556—it possesses broad antimicrobial activity, can stimulate IL-10 production and does not induce cytotoxic effects and hemolysis. Four novel antimicrobial peptides (R10, R11, R12, R13) were designed and demonstrated high antimicrobial activity against *C. acnes* at concentrations well below cytotoxic and hemolytic concentration range. Peptides were found to be selective for *C. acnes* at low concentrations while only being inhibitory against other Gram-positive and Gram-negative bacteria at higher concentrations. This specificity makes peptides designed in this study attractive for further development of anti-acne treatments where a specific pathogen needs to be targeted within the diverse skin microflora.

## Figures and Tables

**Table 1:** Antimicrobial peptide names, corresponding sequences, size, theoretical secondary structure according to APPTEST [52], net charge at pH7, and origin.

Name	Sequence	Size kDa	Secondary Structure	Net Charge at pH7	Reference
RP556	RWCFKVCYKGICYKKCK	2.16	antipar.—β	+5.8	[47]
LZ1	VKRWKKWWRKWKKWV	2.22	α-helix	+8.0	[48]
AA139	GFCWYVCARRNGARVCYRRCN	2.56	antipar.—β	+4.8	[50]
PA13	KIAKRIWKRIWKILRRR	2.32	α-helix	+9.0	[51]
Oligo10	FAKALKALLKALKAL	1.59	α-helix	+4.0	Commercial
R1	FIKKFAKKFKKFIKKFAKFAFAF	2.86	α-helix	+9.0	This work
R10	KKIAKKFWKKFWKFWKIFKK	2.74	α-helix	+10.0	This work
R11	KFCLKFCFKGFCFKACGK	2.11	antipar.—β	+9.7	This work
R12	IAKKFWKKFWKFWKIFKKIA	2.67	α-helix	8.0	This work
R13	IAKKFWPKFWKFWKIFKKIA	2.67	α-helix	7.0	This work
R14	KKIAKKFWKKFWKFWPKFWKIFKK	3.31	α-helix	11.0	This work

**Table 2:** Minimal inhibitory concentrations (MIC) of antimicrobial peptides against selected bacteria, μg/mL.

		MIC μg/mL					
Name	Reference	*E. coli*	*P. aeruginosa*	*K. pneumonia*	*E. faecium*	*S. aureus*	*C. acnes*
RP556	[53]	4.0	2.0	4.0	2.0	1.0	2.0
LZ1	[48]	31.0	8.0	16.0	4.0	2.0	8.0
AA139	[50]	4.0	16.0	8.0	16.0	8.0	63.0
PA13	[51]	31.0	16.0	16.0	4.0	2.0	16.0
Oligo10	Commercial product	16.0	63.0	63.0	16.0	4.0	2.0
R1	This work	8.0	8.0	4.0	16.0	8.0	2.0
R10	This work	2.0	2.0	8.0	4.0	4.0	0.5
R11	This work	125.0	63.0	63.0	16.0	16.0	0.3
R12	This work	8.0	4.0	31.0	16.0	8.0	0.5
R13	This work	4.0	4.0	8.0	16.0	4.0	0.3
R14	This work	16.0	4.0	4.0	8.0	8.0	1.0

**Table 3:** Therapeutic indexes of antimicrobial peptides. Calculations done based on cytotoxicity (IC_50_) in Balb/c 3T3 line and MIC values against listed bacteria.

		Therapeutic Index					
	Reference	*E. coli*	*P. aeruginosa*	*K. pneumonia*	*E. faecium*	*S. aureus*	*C. acnes*
RP556	[47]	96.5	193.0	96.5	193.0	386.0	193.0
LZ1	[48]	5.9	22.9	11.4	45.8	91.5	22.9
AA139	[50]	157.0	39.3	78.5	39.3	78.5	10.0
PA13	[51]	8.5	16.4	16.4	65.5	131.0	16.4
Oligo10	Commercial product	13.6	3.5	3.5	13.6	54.5	109.0
R1	This work	8.6	8.6	17.3	4.3	8.6	34.5
R11	this work	0.9	1.7	1.7	6.7	6.7	356.7
R12	this work	2.0	4.0	0.5	1.0	2.0	32.0
R13	this work	19.8	19.8	9.9	4.9	19.8	263.3
R14	this work	2.2	8.8	8.8	4.4	4.4	35.0

**Table 4:** Therapeutic indexes of antimicrobial peptides. Calculations done based on cytotoxicity (IC_50_) in human primary dermal fibroblast cell line and MIC values against listed bacteria.

		Therapeutic Index					
	Reference	*E. coli*	*P. aeruginosa*	*K. pneumonia*	*E. faecium*	*S. aureus*	*C. acnes*
**R1**	This work	16.01	16.01	32.02	8.0	16.01	64.04
**RP556**	[47]	86.43	172.85	86.43	172.85	345.7	172.85
**Oligo10**	Commercial product	15.1	3.83	3.83	15.1	60.39	120.77
**R10**	this work	19.1	19.1	4.77	9.55	9.55	76.38

**Table 5:** A_π→0_ and π-collapse values of Gram-negative membrane and membrane peptide mixtures derived from π-A isotherms.

Sample	A_π→0_	π Collapse mN/m
Gram-negative membranes	73.1	45.3
Gram-negative membranes + RP556	83.4	43.6
Gram-negative membranes + R10	76.3	43.4

## Data Availability

Not applicable.

## Supplementary Materials

The following supporting information can be downloaded at: https://www.mdpi.com/article/10.3390/antibiotics12030551/s1, Table S1: Minimal inhibitory concentrations (MIC) of antibiotics and non-antibiotic agents against bacteria used in the study, μg/mL.
