# Supplementary material for: Comparative Evaluation of Existing and Rationally Designed Novel Antimicrobial Peptides for Treatment of Skin and Soft Tissue Infections
Source: Antibiotics (Basel). 2023 Mar 10;12(3):551. doi: 10.3390/antibiotics12030551 (PMC10044245; doi:10.3390/antibiotics12030551)
Supplement: Supplementary file 1 [file antibiotics-12-00551-s001.zip › antibiotics-2224218-supplementary.pdf]

Table S1. Minimal inhibitory concentrations (MIC) of antibiotics and non-antibiotic agents against bacteria used in the study, µg /ml.

		E.coli	*P. aeru-ginosa*	*K. pneumonia*	*E. faecium*	*S. aureus*	*C. acnes*
Antibiotics [a]	Azythromycin	-	-	8.0	-	0.5	-
	Claritromycin	-	-	64.0	-	0.25	-
	Amoxiclav	-	-	8.0	-	0.13	-
	Gentamicin	1.0	0.25	0.25	1,0	0.25	-
Non-antibiotic agents [b]	Benzoyl peroxide	31.0	-	-	-	1250.0	80.0
	Chlorhexidine	16.0	1.0	-	-	1.0	1.0

[a] Azithromycin dihydrate (Sigma-Aldrich, St. Louis, MO, USA), clarithromycin (Sigma-Aldrich, St. Louis, MO, USA), amoxiclav (Sandoz, Kundl, Austria; amoxicillin 875 mg, acid clavulanic 125 mg), gentamicin (Sigma-Aldrich, St.Louis, MO, USA). [b] Benzoyl peroxide (Saules Aptieka, Riga, Latvia), chlorhexidine (Saules aptieka, Riga, Latvia).
